# Clinical characterization, physical frailty, and depression in elderly patients with psoriasis from a reference center in Brazil: a cross-sectional study^☆^

**DOI:** 10.1016/j.abd.2023.01.001

**Published:** 2023-08-21

**Authors:** Giovana Viotto Cagnon Brandão, Elizandra Gomes Pereira, Gabriela Roncada Haddad, Luciane Donida Bartoli Miot, Silvio Alencar Marques, Hélio Amante Miot

**Affiliations:** Departamento de Infectologia, Dermatologia, Diagnóstico por Imagem e Radioterapia, Faculdade de Medicina, Universidade Estadual Paulista, Botucatu, SP, Brasil

**Keywords:** Aged, Chronic disease, Immunosenescence, Metabolic syndrome, Psoriasis

## Abstract

**Background:**

There are few studies dedicated to the characterization of the geriatric population with psoriasis, which has particularities in terms of clinical manifestations and therapeutic limitations. As psoriasis is a chronic disease, presenting a higher prevalence with age, the increase in life expectancy in Brazil demands knowledge about the behavior of the disease among the elderly.

**Objectives:**

To characterize elderly people with psoriasis from a tertiary service, from the clinical-epidemiological point of view, presence of comorbidities, physical frailty, and affective impact, and to compare these aspects with adults with psoriasis and elderly people without the disease.

**Methods:**

Cross-sectional study of 64 elderly patients with psoriasis, 64 adults with psoriasis, and 64 elderly patients without the disease. Clinical-demographic aspects, the Beck depression scale, and Skindex-16 were evaluated. Indicators of physical frailty were evaluated in elderly patients: handgrip, sit-to-stand test, fatigue, and weight loss >5%.

**Results:**

In the elderly, the mean age (SD) of psoriasis onset was 44 (10) years, men represented 47% of the sample, the prevalence of arthritis was 22%, and ungual involvement occurred in 72%. Topical corticosteroids were used more often among elderly people with psoriasis (100%) than among adults with the disease (86%), with no difference among other systemic treatments. Diabetes mellitus occurred in 30% of the elderly. Hypertension (59%), dyslipidemia (52%), depression (34%), and fatigue (59%) were more prevalent among the elderly with psoriasis than among the healthy controls.

**Study limitations:**

The study was carried out in a public reference service for patients with psoriasis, all of which were undergoing treatment.

**Conclusions:**

Elderly people with psoriasis from a tertiary service showed greater affective impairment, metabolic comorbidities, and physical frailty than elderly controls.

## Introduction

Psoriasis is a chronic systemic inflammatory disease, immune-mediated by T-lymphocytes (Th1 and Th17 response), which has a multifactorial nature, involving genetic (polygenic) susceptibility, associated with environmental factors such as trauma, medications, infections, and emotional factors.^1^, ^2^

The skin is the main organ affected by psoriasis, although it can affect joints, mucous membranes, and nails, and is associated with other systemic conditions such as Crohn's disease, uveitis, psychiatric disorders, and non-alcoholic fatty liver disease.^3^, ^4^ Patients with psoriasis, from all age groups, have a higher prevalence of metabolic syndrome, characterized by obesity, systemic arterial hypertension, type II diabetes mellitus, dyslipidemia, and an increased risk of developing cardiovascular disease.^5^, ^6^, ^7^

Due to the impact on the quality of life and the induction of systemic inflammation (via TNFα, IL-6, and IL-1), psoriasis is associated with affective disorders, and approximately 30% of the patients have some psychiatric comorbidity (e.g., depression, and anxiety disorder), which compromise work productivity, interpersonal relationships, and increase the risk of suicide.^8^, ^9^, ^10^, ^11^

In Brazil, the prevalence of psoriasis is estimated to be between 1.10% and 1.51%; however, in the elderly, this estimate rises to between 1.71% to 2.84%.^12^ The increase in life expectancy of the population leads to the growth in the prevalence of chronic diseases in the population, among them, psoriasis.

Special considerations should be made regarding assistance to the geriatric population, which tends to have multiple comorbidities and use several drugs concomitantly, maximizing adverse effects and drug interactions. With advancing age, metabolic and antioxidant system changes occur, altering pharmacodynamic and pharmacokinetic processes. Moreover, immunosenescence favors a chronic inflammatory condition that functionally compromises immunity.^13^

There are few systematic studies of psoriasis in the elderly, describing its clinical and therapeutic particularities and its impact on physical frailty.^14^, ^15^ In Brazil, according to Law n. 10,471/033, which deliberates on the Elderly Person Statute, individuals aged 60 years or older are considered elderly. Elderly people are frequently excluded from clinical trials, as they are considered a risk group for adverse treatment events.^13^, ^16^, ^17^, ^18^ Data on the efficacy and safety of systemic and immunobiological drugs in this age group are also scarce.^19^

This study aimed to characterize clinical-demographic variables, presence of comorbidities, physical frailty, and the affective impact on elderly patients with psoriasis treated at a reference dermatology service, as well as compare these aspects in adults with psoriasis and elderly people without the disease.

## Sample and methods

A cross-sectional study (using two control populations) was conducted on 64 patients with psoriasis aged over 60 years of age, 64 patients with psoriasis under 50 years of age, and 64 elderly people without psoriasis. The study was conducted at the Dermatology Outpatient Clinic of Universidade Estadual Paulista, between March 2019 and March 2021.

The sampling was made by convenience, including eligible cases and controls, consecutively, during medical appointments. Patients with psoriasis (elderly and adults) came from the service psoriasis clinic. The elderly controls had other localized dermatological diseases and attended the Dermatology Outpatient Clinic or were accompanying the patients. Individuals with understanding or interpretation difficulties, patients without clinical psoriasis lesions on examination, and patients with other extensive dermatoses were not included in the study.

The project was approved by the Research Ethics Committee of the institution (FMB-Unesp number 3.318.242), and all participants signed the free and informed consent form.

Elderly with psoriasis (EPso), elderly without psoriasis (ESans), and adults with psoriasis (APso) were characterized regarding their demographic, anthropometric characteristics, current treatment, smoking status, alcohol consumption, Beck depression scale score, and main comorbidities.^20^ The elderly (EPso and ESans) were assessed for physical frailty through the sit-to-stand test, handgrip strength test, weight loss, and fatigue.^21^

The Beck scale for self-assessment of depression consists of 21 items that cover the cognitive, affective, behavioral, and somatic components of depression. Each item contains four statements that vary in intensity (0 to 3), being up to the respondent to indicate which of the four statements best describes their symptoms. The final score is obtained through the sum of the 21 items that make up the scale, with the following results: (a) No depression or minimal depression: final scores lower than 11 points; (b) Mild to moderate depression: final scores between 12 and 19 points; (c) Moderate to severe depression: final scores between 20 and 35 points; and (d) Severe depression: final scores between 36 and 63 points.^22^, ^23^ The Beck scale is one of the most used instruments for the evaluation of depressive disorder and its variables, applied in psychiatric and non-psychiatric patients, validated in other countries, and confirmed in terms of reliability and validity criteria.

The sit-to-stand test provides an indirect assessment of the patients physical performance. They were asked to stand up and sit down from a chair of normal height (approximately 48 cm) five consecutive times, without hand support.^24^ Handgrip strength, an indirect criterion of muscle strength, was measured using the handgrip through a manual dynamometer (hand dynamometer DRP-120), with the dominant hand of each patient, and the highest measurement (in kg) of three performed attempts was considered for the study. Weight loss was characterized by a reduction of more than 5% of their weight in the last three years. The assessment of fatigue, according to the Brazilian Consensus on Frailty in the Elderly, was performed by directly asking the patient if they felt “full of energy” at that exact moment.^25^, ^26^

The patients with psoriasis underwent clinical evaluation using the PASI (Psoriasis Area Severity Index) score, BSA (Body Surface Area) score, and PGA (Physician Global Assessment) score for the scalp and body, evaluated by a qualified dermatologist, in addition to the Skindex-16 quality of life questionnaire, validated for Brazilian Portuguese.^27^, ^28^, ^29^

The internal consistency of the Beck scales and the Skindex-16 dimensions were assessed using the McDonald's coefficient ω, and its 95% confidence interval (95% CI). Quantitative and ordinal variables were described as percentages and compared using the chi-square test for trend and contingency table residual analysis (post hoc).^30^ Quantitative variables were described as mean and standard deviation or median and quartiles (p25‒p75) if normality was not verified by the Shapiro-Wilk test.^31^ Correlations between quantitative variables were assessed by Spearman's rho coefficient.^32^ The groups were compared to each other by Poisson (log-linear) regression models, and the effect size was expressed by the prevalence ratio (PR) and its 95% CI.^33^ The data were tabulated in MS Excel 2010 and analyzed using IBM SPSS 25 software. A p-value <0.05 was considered significant.^34^

The sample size was calculated based on a pre-test with 64 participants in each group of elderly people (n = 128), based on the estimate for Poisson (log-linear) regression for the comparison between the variables of physical frailty.^35^

## Results

The main demographic data of the study participants are shown in Table 1. The ESans group stands out with the highest family income, while the APso group showed the highest level of schooling.

**Table 1:** Main demographic data of the studied participants.

Variables		EPso (n = 64)	APso (n = 64)	ESans (n = 64)	p-value
Age in years, mean (standard deviation)		65 (5)	42 (9)	67 (5)	–
Sex, n (%)	Female	34 (53%)	29 (45%)	34 (53%)	0.630
	Male	30 (47%)	35 (55%)	30 (47%)	
Phototype, n (%)	I‒II	5 (8%)	4 (6%)	17 (27%)	0.176
	III‒IV	56 (88%)	57 (89%)	47 (73%)	
	V‒VI	3 (5%)	3 (5%)	‒ (‒)	
Level of schooling, n (%)	Elementary School	49 (77%)^a^	27 (42%)	39 (67%)	**0.001**
	High School	6 (14%)	25 (39%)^a^	11 (17%)	
	Higher Education	6 (9%)	12 (19%)	14 (16%)	
Family income, n (%)^b^	<R$ 1.000,00	9 (14%)	7 (11%)	3 (5%)	**0.004**
	1‒3.000,00	42 (66%)	32 (50%)	37 (58%)	
	3‒5.000,00	13 (20%)	22 (34%)	15 (23%)	
	>5.000,00	‒ (‒)	3 (5%)	9 (14%)^a^	
Current smoking, n (%)		13 (20%)	11 (17%)	10 (16%)	0.840
Alcohol consumption, n (%)		10 (16%)	22 (34%)	15 (23%)	0.050

EPso, Elderly with psoriasis (> 60 years); APso, Adults with psoriasis (<50 years); ESans, Elderly without psoriasis.

a p-value < 0.05 (*post-hoc* analysis).

b R$ 1.00 = Us$ 0.19 (28/Nov/2022).

Regarding the clinical and therapeutic aspects of psoriasis (Table 2), the EPso and APso groups showed comparable clinical parameters; however, they differed in terms of age at the onset of the disease and the use of topical corticosteroids. The classes of immunobiological used by the EPso group were high anti-TNF-α (Etanercept, Infliximab and Adalimumab) and anti-IL-17A (Secukinumab).

**Table 2:** Main clinical and therapeutic characteristics associated with psoriasis in elderly and adults.

Variables		EPso (n = 64)	APso (n = 64)	p-value
Psoriasis onset (age)^a^		44 (13)	28 (11)	**<0.001**
Family history, n (%)		21 (33%)	26 (41%)	0.463
PASI^b^		4 (2‒9)	5 (2‒11)	0.332
	PASI ≥10	13 (20%)	18 (28%)	0.410
BSA^b^		6 (4‒15)	9 (4‒18)	0.402
	BSA ≥10	19 (30%)	26 (41%)	0.267
PGA body^b^		2 (2‒3)	3 (2‒3)	0.734
PGA scalp^b^		1 (0‒2)	1 (0‒2)	0.727
Skindex-16^b^	Symptoms	17 (0‒46)	25 (0‒50)	0.429
	Emotional	23 (2‒63)	27 (11‒73)	0.219
	Functional	0 (0‒35)	7 (0‒40)	0.056
Palmoplantar, n (%)		16 (25%)	21 (33%)	0.436
Ungual, n (%)		46 (72%)	41 (64%)	0.449
	Onycholysis	36 (56%)	28 (44%)	
	Pitting	17 (27%)	23 (36%)	
	Trachyonychia	7 (11%)	3 (5%)	
Arthritis, n (%)		14 (22%)	10 (16%)	0.498
	Hands/wrists	11 (17%)	7 (11%)	
	Knees	4 (6%)	4 (6%)	
Treatment, n (%)	Topical CTC	64 (100%)	55 (86%)	**0.003**
	Calcipotriol	16 (25%)	18 (28%)	0.842
	Methotrexate	30 (47%)	37 (58%)	0.288
	Acitretin	24 (34%)	34 (53%)	0.110
	Phototherapy	11 (17%)	8 (13%)	0.620
	Immunobiologicals	11 (17%)	12 (19%)	0.999
	Cyclosporine	1 (2%)	0% (-)	0.999

EPso, Elderly com psoriasis (> 60 years); APso, Adults with psoriasis (< 50 years); ESans, Elderly without psoriasis; CTC, Corticoid; PASI, Psoriasis area and severity index; BSA, Body surface area; PGA, Physician global assessment – evaluated on the day of inclusion in the study.

a Mean(standard deviation).

b Median (P25–P75).

Among the EPso, five patients (7.8%; 95% CI 1.6 to 15.0) had the disease onset after 60 years of age. When adjusted for the current age and gender, EPso patients with disease onset in old age did not show clinical severity, physical frailty, frequency of comorbidities, or affective impairment that differed from EPso patients with disease onset before the age of 60 years (p < 0.1).

The Skindex-16 inventory showed adequate internal consistency (McDonald's ω) > 0.82 for the three dimensions, with no difference in scores between the groups. The functional dimension showed the best correlation with the Beck score (rho = 0.471; p < 0.001); however, it was not associated with the presence of arthritis (p = 0.131).

Table 3 shows the comparison of the main clinical-demographic elements, affective aspects, and frailty indexes among the groups. There was an increase in the prevalence of comorbidities in the EPso and APso groups, such as hypertension, dyslipidemia, and diabetes. However, when comparing the EPso with the ESans group, in addition to more comorbidities associated with the metabolic syndrome, such as systemic arterial hypertension and dyslipidemia, there was a higher prevalence of fatigue and depression.

**Table 3:** Comparison of the prevalence of the main comorbidities, depression scales and frailty indexes among the groups.

Variables		EPso (n = 64)	APso (n = 64)	ESans (n = 64)	PR1 (EPso × APso)	p-value^1^	PR2 (EPso × ESans)	p-value^2^
BMI in kg/m^2^, mean (SD)		28.3 (4.5)	28.5 (5.6)	28.5 (6.3)	‒	0.394	‒	0.136
	BMI ≥ 30	22 (34%)	26 (41%)	25 (39%)	0.9 (0.7‒1.1)	0.393	1.0 (0.8‒1.1)	0.548
Abdominal circumference in cm, mean (sd)		98,7 (14,4)	100.7 (11.9)	101.6 (14.2)	‒	0.129		0.357
Diabetes mellitus, n (%)		19 (30%)	9 (14%)	14 (22%)	2.0 (1.0‒4.0)	**0.038**	1.1 (0.8‒2.6)	*0.087*
Hypertension, n (%)		38 (59%)	13 (20%)	30 (47%)	2.9 (1.7‒5.0)	**<0.001**	1.3 (1.0‒1.7)	**0.039**
Dyslipidemia, n (%)		33 (52%)	26 (41%)	25 (39%)	1.3 (0.9‒1.8)	0.231	1.4 (1.1‒2.1)	**0.045**
	LDL	22 (34%)	9 (14%)	14 (22%)	1.4 (1.1‒1.9)	**0.020**	1.1 (1.0‒1.3)	0.103
	Triglycerides	8 (13%)	7 (11%)	4 (5%)	1.1 (0.9‒1.3)	0.564	1.2 (1.0‒1.5)	0.107
	Both	3 (5%)	10 (16%)	6 (9%)	0.7 (0.5‒0.9)	**0.006**	0.9 (0.7‒1.2)	0.552
Cardiovascular event, n (%)		7 (11%)	4 (6%)	7 (11%)	1.0 (1.0‒1.1)	0.313	1.0 (0.9‒1.1)	0.733
Beck scale, median (p25‒p75)		6.0(3.0‒11.5)	6.5 (2.0‒12.0)	5.0 (2.0‒8.0)	‒	0.779	‒	**<0.001**
	Beck ≥10	22 (34%)	20 (31%)	13 (20%)	1.0 (0.6‒1.7)	0.890	1.7 (0.9‒3.1)	*0.068*
Physical frailty indexes in the elderly								
Handgrip in kg, median (p25‒p75)		3.5 (0.0‒10.0)	‒	5.0 (0.0‒10.0)	‒	‒	‒	*0.094*
Fatigue, n (%)^a^		38 (59%)	‒	23 (36%)	‒	‒	1.7 (1.2‒2.4)	**0.003**
Sit-to-stand test, n (%)^b^		60 (93%)	‒	59 (92%)	‒	‒	1.0 (0.9‒1.1)	0.895
Weight loss >5% (%)		17 (27%)	‒	12 (19%)	‒	‒	1.5 (0.8‒2.8)	0.239

EPso, Elderly with psoriasis (> 60 years); APso, Adults with psoriasis (<50 years); ESans, Elderly without psoriasis; PR, Prevalence ratio.

1 Comparison between EPso and APso (gender-adjusted data).

2 Comparison between EPso and ESans (data adjusted for sex and age).

a Fatigue: defined by those who answered negatively to the question “Do you feel full of energy”.

b Complete Sit-to-stand test: able to sit on and stand up from the chair five consecutive times.

The Beck scale showed high internal consistency and the McDonald's coefficient ω was 0.86 (95% CI 0.83 to 0.89). Items B1 (feeling of sadness) and B15 (ability to work) were prominent (p < 0.05) in the difference between the EPso and ESans groups.

In patients with psoriasis, those with arthritis had lower mean handgrip strength (5.1 vs. 10.0 kg; p = 0.030); however, arthritis was not a determinant of performance in the sit-to-stand test or fatigue assessment (p > 0.760). As for the Beck scores, they were higher for the elderly who reported fatigue and weight loss (p < 0.005).

## Discussion

In this Brazilian sample of patients treated at a reference center, elderly people with psoriasis had an average age of onset of psoriasis after the fourth decade, with no gender preference, and the clinical characteristics, in addition to the impact on quality of life, did not differ from adults with psoriasis attended at the same clinic. Despite this, they had a higher prevalence of depressive symptoms, elements of physical frailty, and metabolic syndrome parameters than elderly people without psoriasis.

About 10% of psoriasis cases seen in dermatology practice are elderly patients; however, this prevalence is increasing and is expected to rise with the aging of the population.^13^, ^36^ In addition to increased life expectancy, the population access to information about the disease, and to the health system to confirm the diagnosis, is another factor that may have led to an increase in the incidence of psoriasis as a whole in recent decades.

The elderly patients with psoriasis can be divided into those who had the onset of the disease during senescence (after 60 years of age), which corresponds to 3% to 13% of cases, and those who started it earlier (under 60 years of age). Psoriasis with onset after the age of 60 is expected to have a milder clinical picture, with lower rates of BSA, ungual and joint involvement, compared to patients with an earlier onset. However, studies dedicated to its characterization are rare, as the representation of the geriatric population in clinical trials is still scarce.^37^, ^38^ Only 15% of this population develops moderate to severe psoriasis and requires systemic treatments. In the present sample, the authors did not observe any difference between the distribution of PASI, BSA scores, body/scalp PGA, palmoplantar or nail involvement, and arthritis between the two groups of patients with psoriasis (EPso and APso).

In a sample of 156 elderly Iranians with psoriasis, demographic characteristics, comorbidities, age of disease onset, predominant clinical form, disease severity, and therapeutic approaches were evaluated.^36^ The most common clinical type of the disease was the plaque type (73.1%), followed by the pustular type (10.9%), and the most frequently affected site was the extremities (84.6%). Scalps, nails, and joints were involved in 26.9%, 40.3%, and 19.2% of the assessed patients. The mean PASI score was 5.0 ± 5.5. Hypertension (25%) and diabetes mellitus (16%) were the most prevalent diseases. Regarding patients therapeutic approach, most were treated topically (81.7%), and among systemic treatments, methotrexate and acitretin were the most frequently used ones, accounting for 74.4% and 60.3% of the patients. Men had a lower mean age of disease onset (51.8 years) compared to women (59.2 years). Patients with joint involvement had a lower mean age (65.2 ± 6.3 years) when compared to those without this involvement (69.2 ± 7.4 years).^39^

Age is an additional risk factor for hypertension, dyslipidemia, depression, and type 2 diabetes. In the present sample of elderly people with psoriasis, these parameters were more prevalent than in the elderly controls. In fact, when compared to healthy controls, psoriasis patients have an increased prevalence of dyslipidemia, hypertension, coronary artery disease, type 2 diabetes, and increased Body Mass Index (BMI).^35^, ^40^, ^41^, ^42^ These elements may corroborate the higher cardiovascular mortality among patients with psoriasis.^43^, ^44^

In controlled studies, the odds ratio for dyslipidemia in patients with psoriasis was 2.5 (95% CI 1.7‒3.3), diabetes mellitus was 1.8 (95% CI 1.6‒2.0) and systemic arterial hypertension was 1.6 (95% CI 1.4‒1.8) when compared to healthy patients.^40^, ^45^, ^46^, ^47^, ^48^, ^49^ It is noteworthy that these elements may also result from systemic therapies for psoriasis, as in the case of retinoids, which can raise triglyceride levels, as well as the weight gain seen in users of anti-TNF immunobiological, and hypertension among users of cyclosporine.^50^, ^51^ In the present study, the increase in prevalence of dyslipidemia and hypertension was relevant in the EPso group, when compared to ESans; however, this result was not confirmed in relation to diabetes.

Treating psoriasis in elderly patients can be challenging. Most of the time, this population suffers from multiple comorbidities and uses several medications, in addition to progressive functional impairment of the immune system, which increases susceptibility to infections, as well as self-reactivity and adverse effects to existing therapies. Moreover, logistic issues, such as depending on caregivers for activities of daily living, can influence the therapeutic choice. Thus, systemic and immunobiological therapies can be postponed and topical treatments be used as the first option, especially topical corticosteroids, not because of how easily they can be administered to the patient, but rather because of the risk of possible adverse events and drug interactions that other therapies would pose.^13^, ^20^ The present results corroborate this indication since there was a higher frequency of treatment with topical corticosteroids in the EPso (100%) than in the Apso group (86%).

Also, topical corticosteroids should be used with caution in this population, taking into account the changes that occur in aging skin, as well as the increased risk of cutaneous side effects such as purpura, atrophy, hematomas, skin infections, and tachyphylaxis.^37^, ^47^ It must be stressed that therapeutic expectations in the elderly should be the same when compared to younger patients. Conventional systemic treatments, such as methotrexate and acitretin, are often contraindicated in the geriatric population due to the side effect profile and higher rates of drug interactions, taking into account the multiple comorbidities and the greater number of drugs used by these patients. Phototherapy may not be convenient because elderly skin already undergoes changes inherent to aging and thus becomes more susceptible to possible side effects, such as skin cancer or hypersensitivity. Regarding immunobiologicals, preliminary data regarding their safety and effectiveness are optimistic for use in this subgroup of patients.

The impact of chronic dermatoses, such as psoriasis, on psychological and mental health is an important consideration, given the implications of the disease on the individuals well-being and treatment. Psychological stress is commonly considered a trigger for worsening psoriasis, and the chronic contingency that the dermatosis inflicts leads to the development of affective symptoms.^52^ Patients with psoriasis have an increased prevalence of depression and anxiety, in addition to suicidal ideation,^53^, ^54^, ^55^ data that is corroborated by the present study, which showed a higher percentage of depressive symptoms in elderly people with psoriasis when compared to elderly people without the disease. The high prevalence of depressive symptoms identified in adults with psoriasis is also noteworthy. Therefore, attention should be paid to the identification and treatment of psychiatric comorbidities in these patients, in addition to the fact that mental disorders can be a factor for non-adherence to treatment.

Frailty is defined as an energy decline syndrome in the elderly and is based on changes intrinsic to aging, such as sarcopenia and immune dysfunction, and extrinsic ones, such as disease, immobility, and decreased energy.^22^ The frail elderly have a compromised daily ability to deal with acute stress, making them more susceptible to aggressors and dependent on others. Frailty is manifested by fatigue, decreased walking speed and handgrip strength, weight loss, and low levels of physical activity.^22^ The authors evaluated these indicators and identified higher levels of fatigue in the EPso group compared to the ESans group, corroborating the fact that psoriasis would be an additional factor of frailty in elderly people with the disease. The presence of arthritis also adds frailty to elderly people with this condition, limiting their functionality, since the geriatric patients with arthritis had lower handgrip strength during the assessments. Likewise, depression scores were associated with patient frailty and increased functional impairment.

This study has natural limitations associated with the population attended at a single center public institution, and patients undergoing treatment in a secondary service, which, on the other hand, ensured a certain sample homogeneity and allowed assessment of factors in the groups. Severity indexes are difficult to characterize, as all patients were undergoing treatment. More studies are needed to evaluate this age group, since the increase in the prevalence of chronic diseases, including psoriasis, has accompanied the increase in life expectancy of the general population.

## Conclusion

There was no difference between the clinical presentation, ungual and scalp involvement, or the presence of arthritis, between the assessed groups of elderly people and adults with psoriasis. Elderly people treated at a reference center had higher rates of depressive symptoms, metabolic comorbidities, and physical frailty than the healthy elderly controls.

## Financial support

FAPESP (project number 19/06684-5).

## Authors' contributions

Giovana Viotto Cagnon Brandão: Design of the study; data collection; drafting and editing of the manuscript; review and approval of the final version of the manuscript.

Elizandra Gomes Pereira: Data collection; drafting and editing of the manuscript; review and approval of the final version of the manuscript.

Gabriela Roncada Haddad: Data collection; drafting and editing of the manuscript; review and approval of the final version of the manuscript.

Luciane Donida Bartoli Miot: Design of the study; review and approval of the final version of the manuscript.

Silvio Alencar Marques: Review and approval of the final version of the manuscript.

Hélio Amante Miot: Conception of the study; analysis of results; drafting and editing of the manuscript; review and approval of the final version of the manuscript.

## Conflicts of interest

None declared.
